# Society Meetings

**Published:** 1904-05

**Authors:** 


### SOCIETY MEETINGS.

The Buffalo Acadamy of Medicine held meetings during the months of March and April as follows:

*Section on Surgery.*—Tuesday evening, March 1.—Program: A consideration of the question of drainage in cases of acute appendicitis with spreading peritonitis, Lucius W. Hotchkiss, New York, president of the New York Surgical Society.

*Section on Medicine.*—Tuesday evening, March 8.—Program: Malignant endocarditis, Henry R. Hopkins; discussion by Chas. G. Stockton and Geo. W. York.

*Section on Pathology.*—Tuesday evening, March 15. Program: The serum test for human blood in medico-legal cases, with demonstration, Herbert U. Williams. Specimens and cases will be exhibited by DeLancey Rochester, A. L. Woehnert, C. A. Bentz, W. H. Mosher and Carroll Roberts.

*Section on Obstetrics and Gynecology.*—Tuesday evening, March 22. Program. Sterility and its treatment, Herman E. Hayd; discussion by S. Y. Howell, L. G. Hanley, L. Schroeter, and P. W. Van Peyma.

*Section on Ophthalmology, Otology, Laryngology and Rhinology.*—Tuesday evening, March 29. Program: Diseases of the accessory sinuses of the nose, especially of the maxillary antrum, George L. Richards; discussion will be lead by Frank W. Hinkel and W. Scott Renner.

*Section on Surgery.*—Tuesday evening, April 5. Program: Syphilitic gummata of skin and subcutaneous tissues, illustrated by stereopticon, Grover W. Wende.

*Section on Medicine.*—Tuesday evening, April 12. Program: Enuresis nocturna, Julius Ullman; discussion by Irving M. Snow; Mental changes of old age, Herman G. Matzinger; discussion by Floyd S. Crego.

*Section on Pathology.*—Tuesday evening, April 19. Program: Analysis of stomach contents and other physiological fluids, with theoretical deductions regarding the roll played by chlorides and other salts in human metabolism, G. H. A. Clowes; discussion by Allen A. Jones; Demonstration of transplantation carcinomata in mice, H. R. Gaylord.

---

The sixty-eighth annual meeting of the Medical Society of the County of Chemung will be held at Elmira, on Tuesday, May 17, 1904, beginning at 2.30 o'clock p. m., under the presidency of Dr. Hamilton D. Wey, Elmira. Among the papers to be read are the following: (1) Annual address of the president, The present status of vaccination; (2) urinalysis in pregnancy, Dr. Anna M. Stewart; (3) deferred operation in the repair of lacerated perineum, Dr. A. H. Baker; (4) asepsis in the obstetric chamber, Dr. William Warren Potter, Buffalo; (5) accurate determination of blood pressure as an aid to diagnosis, by Dr. W. Harry Glenny, Buffalo.

---

The American Academy of Medicine will hold its twenty-ninth annual meeting at the Shelburne, Atlantic City, beginning on Saturday, June 4, at 11 a. m., and continuing through Monday, June 6, 1904, under the presidency of Dr. John B. Roberts, of Philadelphia. Dr. Charles McIntire, of Easton, Pa., is the secretary.

---

The Western New York Homeopathic Medical Society held its twentieth annual meeting at the Hotel Iroquois, Buffalo, April 8, 1904, under the presidency of Dr. D. H. Arthur, Gowanda.

---

The Lake Erie Medical Society held its regular quarterly meeting at Dayton, April 8, 1904. Dr. S. S. Bedient is the president, and Dr. B. E. Smith, Angola, is the secretary. The following program was observed: Treatment of gonorrhea, Joseph Rieger, Dunkirk; Some inflammations of pelvis and abdomen, McDonald Moore, Fredonia; Hematmisis, due to back pressure from contracted liver, A. L. Benedict, Buffalo; Pyemia, Walter H. Vosbury, Dunkirk.

